# Starting in your mental pole position: hypnosis helps elite downhill Mountainbike athletes to reach their optimal racing mindset

**DOI:** 10.3389/fpsyg.2024.1334288

**Published:** 2024-05-22

**Authors:** Nina Hoffmann, Jana Strahler, Barbara Schmidt

**Affiliations:** ^1^Institute for Psychology, Friedrich-Schiller-University of Jena, Jena, Germany; ^2^Department of Sport and Science, Sport Psychology, Albert Ludwig University Freiburg, Freiburg, Germany; ^3^Institute of Psychosocial Medicine, Psychotherapy and Psychooncology, Jena University Hospital, Jena, Germany

**Keywords:** hypnosis, competitive anxiety, self-confidence, heart rate variability, Mountainbike downhill

## Abstract

**Introduction:**

Downhill Mountain Biking is an extreme sport requiring high mental strength to perform on the best level in a competition with only one run to win the race. The substantial challenge here is to control automatic processes like competitive anxiety and stress. Hypnosis can address these automatic processes. We developed and evaluated a hypnosis audio-intervention to activate the optimal racing mindset.

**Methods:**

In our study, 19 elite Downhill Mountainbike athletes registered at two consecutive races of the IXS Downhill Cup. After the first race, athletes listened to the hypnosis audio-intervention. In this intervention, we instructed the athletes how to activate their optimal mental state before the second race. At both races, we measured competitive anxiety, stress, self-confidence, state resilience, and flow with validated questionnaires and assessed resting heart rate variability as physiological measure of resilience.

**Results:**

Race-related somatic anxiety and subjective stress decreased significantly while self-confidence increased significantly from first to second race after athletes listened to the hypnosis. Heart rate variability was significantly increased at the second race indicating elevated vagal activity. When comparing race results of our participants to a control group of other elite athletes competing in the races but not listening to the hypnosis, we found that our study participants generally performed better in both races.

**Conclusion:**

The study shows that our hypnosis intervention was effective in reducing competitive anxiety and stress while increasing perceived resilience and self-confidence: After a self-administered hypnosis session, athletes were able to improve automatic processes responsible for putting them in their mental pole position.

## Introduction

Downhill Mountain biking is a subdiscipline of mountain biking and a high-risk sport (Becker et al., 2013). Races are in an individual time-trial format, with cyclists starting at intervals. The aim is to complete a downhill race track in the fastest time possible on a defined route. Stress and anxiety levels are high on race day as athletes only get one single run to win the race. Tracks are physically and psychologically demanding including technical features, high speed sections and large jumps. A high level of self-confidence is one of the most important variables for Downhill Mountainbike speed (Chidley et al., 2015). Hence, not only physical fitness but mental strength is crucial for peak performance outcomes.

To achieve peak performance in a competition, athletes have to learn how to get into an optimal performance state (Privette, 1981; Ruiz et al., 2015). The “Individual Zone of Optimal Functioning” (IZOF) proposes an optimal performance state depending on athlete’s personality, experience and the type of sport (Hanin, 2000). According to the Yerkes-Dodson law of an inverted U relationship between arousal and performance, low levels of arousal lead to boredom, while high levels of arousal cause anxiety, both resulting in deteriorated performance (Yerkes and Dodson, 1908). Therefore, we aim for an optimal level of arousal. Especially an increase of anxiety induces stress in the athlete and is associated with an elevated risk for injuries (Ivarsson et al., 2017). Finally, self-confidence increases with a growing ability to control arousal and anxiety (Liggett, 2004) and reveals a consistent relationship with performance (Craft et al., 2003).

Reaching peak performance is closely related to experiences of a flow state. The flow state is described as a positive psychological state of intense focus, concentration, control, and self-confidence. It typically occurs when an athlete’s perceived skill matches the perceived challenges of a task (Nakamura and Csikszentmihalyi, 2009). A high level of self-confidence is associated with reaching flow state (Koehn, 2013). The experience of flow is crucial for competitive athletes as it facilitates the peak performance state (Koehn, 2013). A downhill Mountainbike descent provides an optimal setting for athletes to induce the core elements of flow (Taylor and Carr, 2021). One element of our intervention is thus the re-definition of the competition as an opportunity to feel great and again find back to the original motivation that made them choose their sport.

Many of the described experiences during flow state and optimal performance can also be experienced under hypnosis (Pates and Maynard, 2000). Hypnosis is a specific state of consciousness characterized by focused attention that increases the capacity to respond to suggestions (Elkins et al., 2015). Most hypnosis paradigms use specific suggestions to modify perception and behavior. Numerous studies have shown that a specific suggestion is associated with suggestion-specific neuronal, behavioral, and psychological effects (Schmidt et al., 2017; Franz et al., 2020; Franz et al., 2021; Schmidt et al., 2021). Therefore, a hypnotic trance depicts an effective way for athletes to reach their IZOF outside a competition (Meiss, 2016). A post-hypnotic trigger implemented during hypnosis and connected to the suggested feelings allows an automatic reactivation outside the hypnotic state. That means, participants experience the hypnosis session and all suggested feelings and then tie these feelings to an eliciting trigger that activates these feelings again outside of the hypnosis session. To illustrate this, participants can tie the feeling of safety to a piece of paper where they write the letter S for safety on it during hypnosis. When they are facing a challenge afterwards, they can use the S paper to elicit the feeling of safety again (Böhmer and Schmidt, 2021; Schmidt et al., 2024a). In another study, we tied the feeling of remembering easily to a piece of paper where participants write the letter E for easy remembering on during hypnosis. When they had to remember the words they just learned, they could use the E paper to make it easier to remember (Schmidt et al., 2024a). In our study, we let participants of the hypnosis group listen to an audio-recorded hypnosis session at home before the race started. We suggested athletes during this hypnosis session that they feel in their optimal mental state to perform on their next race. Then, we established an individual eliciting trigger like pulling the brakes of the bike before the race starts to elicit the feeling of an optimal race. That means the athletes can activate their mental pole position without anyone noticing it on race day just by the routinely movements.

Several studies have investigated sport performance after different hypnosis interventions or a single hypnosis intervention. In controlled studies and single-case design studies, positive effects of hypnosis on athletic performance have been found in different sports, for instance in basketball (Schreiber, 1991; Pates et al., 2002), golf (Pates and Maynard, 2000; Pates et al., 2001b), archery (Robazza and Bortoli, 1995), badminton (Pates and Palmi, 2002) and weightlifting (Lee Howard and Reardon, 1986). However, very few studies have investigated the effect of hypnosis on the optimal performance state in competitions to date (Schreiber, 1991; Lindsay et al., 2005; Pates, 2013; Mattle et al., 2020). No studies have investigated the effects of a hypnosis intervention in mountain biking. Finally, hypnosis interventions seem to be more effective for dexterity, coordination and technical sports (Milling and Randazzo, 2016). Downhill Mountainbike is a sport that requires automatic control of precise movements, fine motor skills, timing and decision making. Thus, we expect a hypnosis intervention to be a successful method to induce an optimal performance state and flow in a downhill Mountainbike race.

Arousal and the experience of flow can be measured via self-reported questionnaires. Additionally, heart rate variability (HRV) as a biomarker provides an inside of the athlete’s physiological changes caused by emotional and cognitive processes (Laborde et al., 2018; Balaban, 2019). It reflects continuous fluctuations in beat-to-beat intervals and presents parameters to quantify the sympatho-vagal balance of the autonomic nervous system (ANS). Changes in HRV can be used to assess anxiety-state changes and performance (Murray and Raedeke, 2008; Mateo et al., 2012; Morales et al., 2013). The relationship between competitive anxiety measured by the Competitive State Anxiety Inventory-2 Revised (CSAI-2R) and HRV has been found in Bicycle Moto Cross (BMX; (Mateo et al., 2012)) and judo athletes (Morales et al., 2013). Therefore, HRV provides an economic tool to assess arousal in a competition (Murray and Raedeke, 2008).

In the present study, we developed an audio-recorded hypnosis intervention to induce an optimal performance state in downhill Mountainbike athletes outside of their competition. We selected athletes that participated in two consecutive official races. Participants in the hypnosis group received the hypnosis intervention between both races, the control group did not. We assessed anxiety, self-confidence, stress and HRV in the hypnosis group to compare their mindset in the two races to test if the hypnosis intervention successfully elicited an optimal mental pole position in the second race.

**Figure 1:**
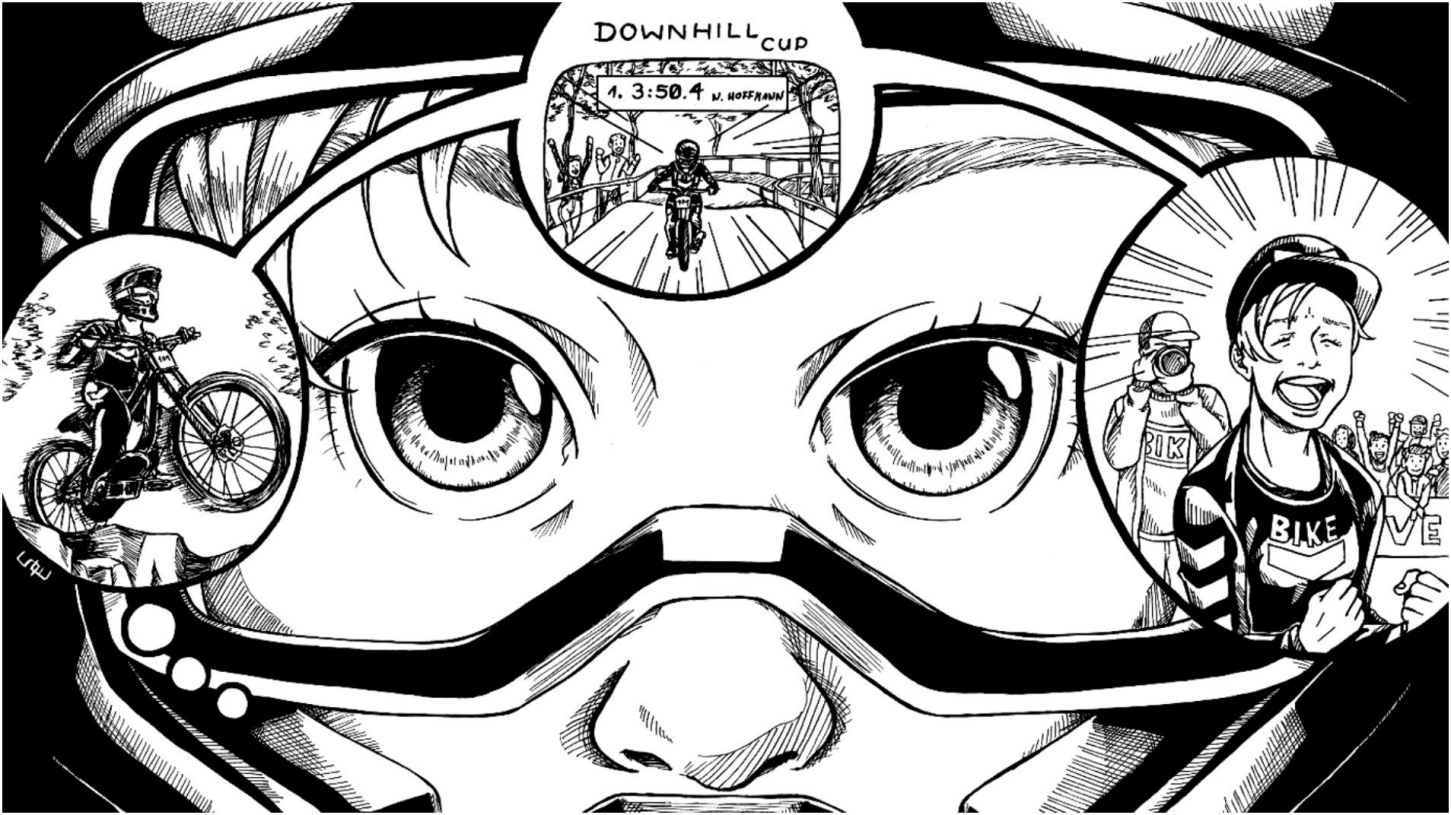
Visualization of the hypnosis intervention. Downhill Mountainbike athletes imagine an optimal race, their finish and most importantly, the feeling of success after an optimal race. These mental images are associated with a personal trigger, so the athletes can activate their optimal performance mode before the race starts.

Figure 1 visualizes the content of our hypnosis intervention. To explore the effects of the intervention, we compared self-reported competitive anxiety, self-confidence, stress, resilience, and flow in two races before and after the hypnosis audio-intervention. Additionally, we recorded HRV data in two races before and after the intervention to assess the physiological state of the athletes before the race. We expected reduced feelings of competitive anxiety and stress before the race, as well as enhanced self-confidence and resilience. Furthermore, we predicted a higher flow state level during the race and expected changes in the parameters of HRV associated with parasympathetic activity. Finally, we expected race performance to be enhanced.

## Methods

### Participants

Based on the effect size of post-hypnotic suggestions on subjective ratings in previous studies conducted in our laboratory (Böhmer and Schmidt, 2021; Schmidt et al., 2024a,b), we performed a power analysis with G*power resulting in a required sample size of 17 participants to detect an effect of *d* = 0.7 in a within-subjects design ((Faul et al., 2007); *d* = 0.7, *alpha* = 0.05, *power* = 0.85). Via social networks (Instagram), we recruited downhill Mountainbike athletes who participated in two consecutive downhill Mountainbike races. Our final sample consisted of 19 downhill Mountainbike athletes (5 female) all competing on national level who voluntarily took part in the study. The athletes’ age ranged between 15 and 36 years (*M* = 20.4, SD = 5.57) and they trained between 2 and 20 h weekly (*M* = 13.05, SD = 4.08). They had between 0.5 and 7 years of race experience (*M* = 3.88, SD = 2.08) and raced between 2 and 13 races per year (*M* = 7.25, SD = 2.99). Half of the athletes (*N* = 10) had experience in international competition with five athletes on World Cup level. Two athletes had experience with mental training. None of them had experience with hypnosis.

The study was approved by the local ethics committee of the Jena University Hospital, with reference number 2022-2568-BO. All athletes filled an informed consent statement and then completed an online questionnaire prior to the races to gather demographic data, training frequency, race experience, hypnosis experience and a trait anxiety score, via the “Wettkampf-Angst Inventar Trait” (WAI-T; Brand et al., 2009). All further data were collected at the IXS Downhill Cup, which is the highest national series of downhill Mountainbike racing in Germany. Athletes can compete at this series either in an open category (recreational category, accessible to all riders) or a license category (elite category, only accessible with a race license). All athletes except of two raced in the license category.

### Experimental design

**Figure 2:**
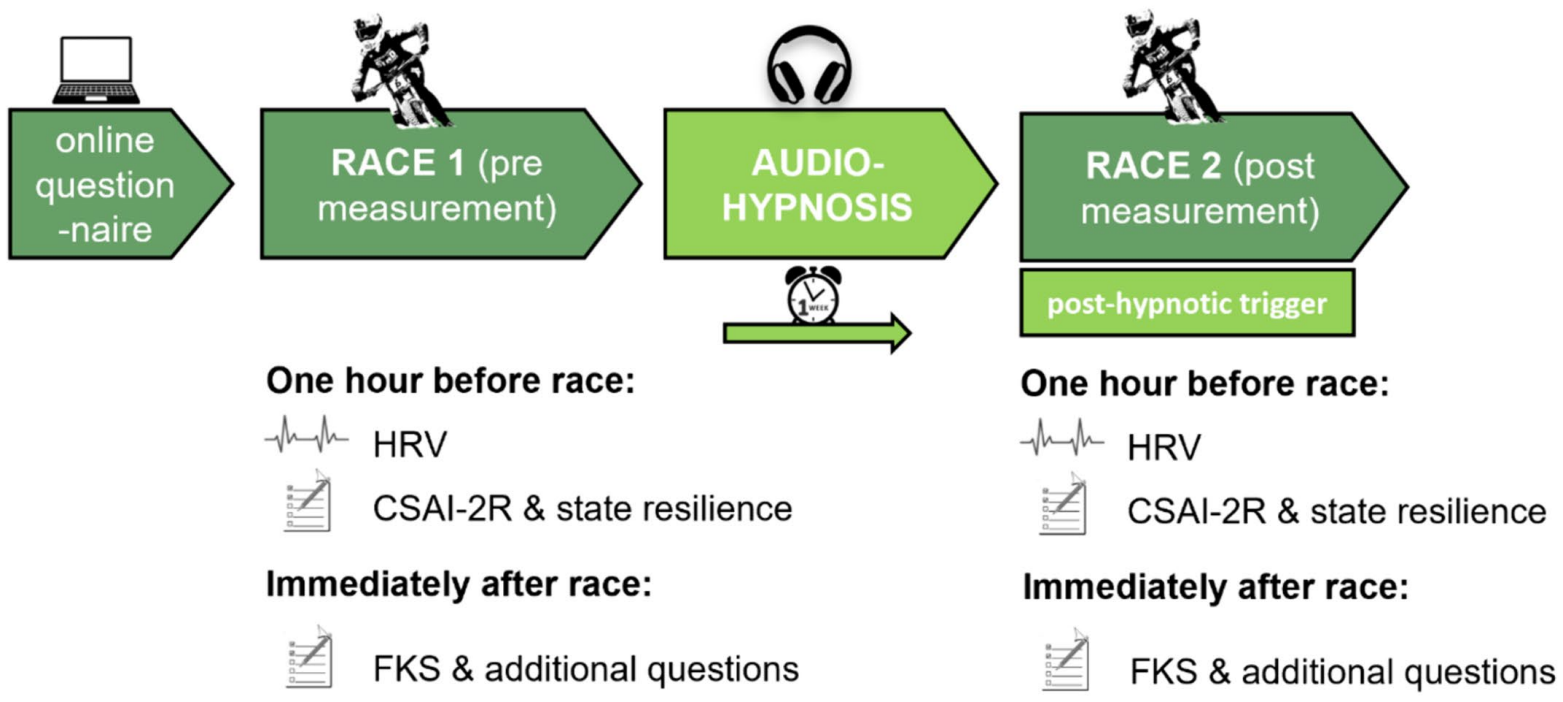
Experimental design and study procedure.

We used a within-subject design with a pre and post measurement of the same participants (Figure 2). The first race was the pre measurement (race 1) to measure baseline performance. After that, participants listened to the hypnosis audio-intervention, followed by a post measurement (race 2). The pre measurement took place at the IXS Downhill Cup in Willingen 2022. One hour before the race, athletes filled in a questionnaire, and we measured heart rate variability. Immediately after the race, athletes filled in a second questionnaire. After the pre measurement, athletes listened to the audio-hypnosis at least once until the post measurement during the next race. The post measurement took place at the IXS Downhill Cup in Winterberg 2022 1 week after the first race.

### Race performance measurement

In a downhill Mountainbike race, time to complete the race track is the dependent variable to assess performance. The athlete with the shortest time to complete the track wins the race. In our study, placement and time behind the winner of each category provided the assessment for performance. As a control group, we used the official race tables of both races and compiled a group consisting of athletes who participated in both races (pre and post measurement) but did not officially participate in our study and accordingly did not receive the hypnosis audio-intervention. Our hypnosis group included all athletes who participated in both races (pre and post measurement) and received the hypnosis audio-intervention. We compiled a new placement table for each race including only athletes of hypnosis and control group to compare their performance between races. Time behind the winner of each category was set in relation to total race time of the winner of each category (variable TIME) to achieve a fair proportional ratio of performance between both race as track length differed.

### Competitive anxiety, self-confidence, state resilience and flow measurement

One hour before the race run each athlete filled the German version of the Competitive State Anxiety Inventory-2 Revised (CSAI-2R; (Cox et al., 2003)) to measure competitive anxiety and self-confidence. In addition, a state resilience questionnaire (Schwerdtfeger and Dick, 2019) was filled to measure state resilience and subjective stress before the race.

Immediately after the race each athlete filled the Flow Kurzskala (FKS; (Rheinberg et al., 2019)). Moreover, we asked questions regarding the subjective assessment of the athlete’s race run performance. In the post measurement athletes additionally rated the hypnosis audio-intervention.

### HRV recording and processing

Resting heart rate variability (HRV) was measured 1 h before the race run to assess physiological stress levels in each athlete. A Polar H10 chest strap (Speer et al., 2020) was connected to the Polar Sensor Logger Application (Jukka Happonen, available in Google Play Store) to record HRV data. Athletes were sitting on a chair, instructed to be quiet and calm. After 1 min of resting the 5 min measurement started. All athletes were asked to voluntary wear the chest strap during their race run to collect additional HRV data. This data has only been used for explorative analysis.

We analyzed all HRV data with Kubios HRV Standard version 3.5.0 Analysis Software (Tarvainen et al., 2013). The middle 4 min of the recorded resting HRV were applied for each athlete to reduce artifacts. For each dataset with more than 5% artifacts low threshold smoothing was applied. For HRV time domain analysis, we analyzed heart rate (HR), mean RR interval (MEANRR) and root mean square of successive differences (RMSSD). For HRV frequency domain analysis, we applied autoregressive modeling (Laborde et al., 2017). The normalized power (n.u.) of high frequencies (HF_nu_) and the ratio of low frequencies to high frequencies power (LF/HF ratio) were analyzed to estimate the sympatho-vagal balance of the ANS.

### Hypnosis and establishment of post-hypnotic trigger

We provided the hypnosis intervention as an audio-intervention. The first part of the original recording of the hypnosis audio-intervention is accessible via this link^1^. Nina Hoffmann, the first author of the study and herself a well-known MTB athlete, recorded the audio-intervention. As Nina Hoffmann won four times the German national championship and became vice world champion in downhill Mountainbike, she was perceived as a very competent person and achieved high rapport in our participating athletes. During the week between both races, we instructed the athletes of the hypnosis group to listen to our hypnosis intervention. They were encouraged to find a quiet room with a comfortable seat and listened to the audio tape on their own. To induce a hypnotic state, we followed the hypnosis induction of the Stanford Hypnotic Susceptibility Scale, Form C (Weitzenhoffer and Hilgard, 1962). Afterwards, we used a time progression method that establishes positive imaginations of the future. The athletes were suggested to imagine their next race in an optimal way. Therefore, athletes thought at first about the intense positive feelings of joy, luck, pride, and success in the finish area after completing an optimal race run. From there we went into their race run imagined in an optimal way always evoking the positive feeling from the finish area. Finally, we imagined the last minutes before the race start when nervosity is the highest and again, transferred this arousal into the positive feeling from the finished area. At the end, these feelings were tied to an individual personal anchor. We counted from 1 to 10 to enhance the positive feelings one last time and then encouraged the athlete to find a personal ritual or routine that could easily be established in their warm-up routine at the next race. We gave some examples like pedaling backwards on the bike, pulling the brake lever, listening to a certain song, or slamming on the chest. Athletes were allowed to choose any personal anchor they had in mind. This allows a reactivation outside the hypnotic state, for example before their next race start. Finally, athletes were led out of the hypnotic state. Athletes were allowed to listen to the audio-hypnosis as often as desired, but at least once. The audio-hypnosis session lasted about 35 min.

### Statistical analysis

Statistical analysis was performed using R version 4.2.2 (R Core Team, 2022). We compared the performance data, self-reported data and HRV data of baseline and post measurements conducting paired-samples *t*-tests. Race performance of hypnosis and control group has been compared conducting Welch’s *t*-tests. In addition, we analyzed the evaluation of the established post-hypnotic trigger. To quantify effect sizes Cohen’s *d* for within-subjects *t*-tests was computed. For all analyses, a *p*-value of <0.05 was considered statistically significant.

## Results

### Race performance data

Athletes of the hypnosis group (*N* = 19) showed significantly better performance compared to athletes of the control group (*N* = 108) at both races. Specifically, we found better placements in the hypnosis group (first race: *t*(52.29) = 4.15, *p* < 0.001, second race: *t*(38.01) = 4.4, *p* < 0.001) and faster race times, assessed via our TIME variable (first race: *t*(59.9) = 2.21, *p* = 0.03, *d_z_* = 0.34; second race: *t*(43.42) = 3.03, *p* = 0.004, *d_z_* = 0.49).

The performance of both groups did not improve from first to second race. There was no improvement in placement (*t*(17) = 0.62, *p* = 0.54) and TIME in the hypnosis group (*t*(17) = 0.35, *p* = 0.73). The performance results of the control group did not improve (placement: *t*(107) = 0.28, *p* = 0.78; TIME: *t*(107) = 0.52, *p* = 0.6). There were no significant changes in the hypnosis group in self-reported mistakes (*t*(18) = 0.37, *p* = 0.72) and crashes (*t*(18) = 1, *p* = 0.33) during the race run.

### Competitive anxiety and self-confidence

**Figure 3:**
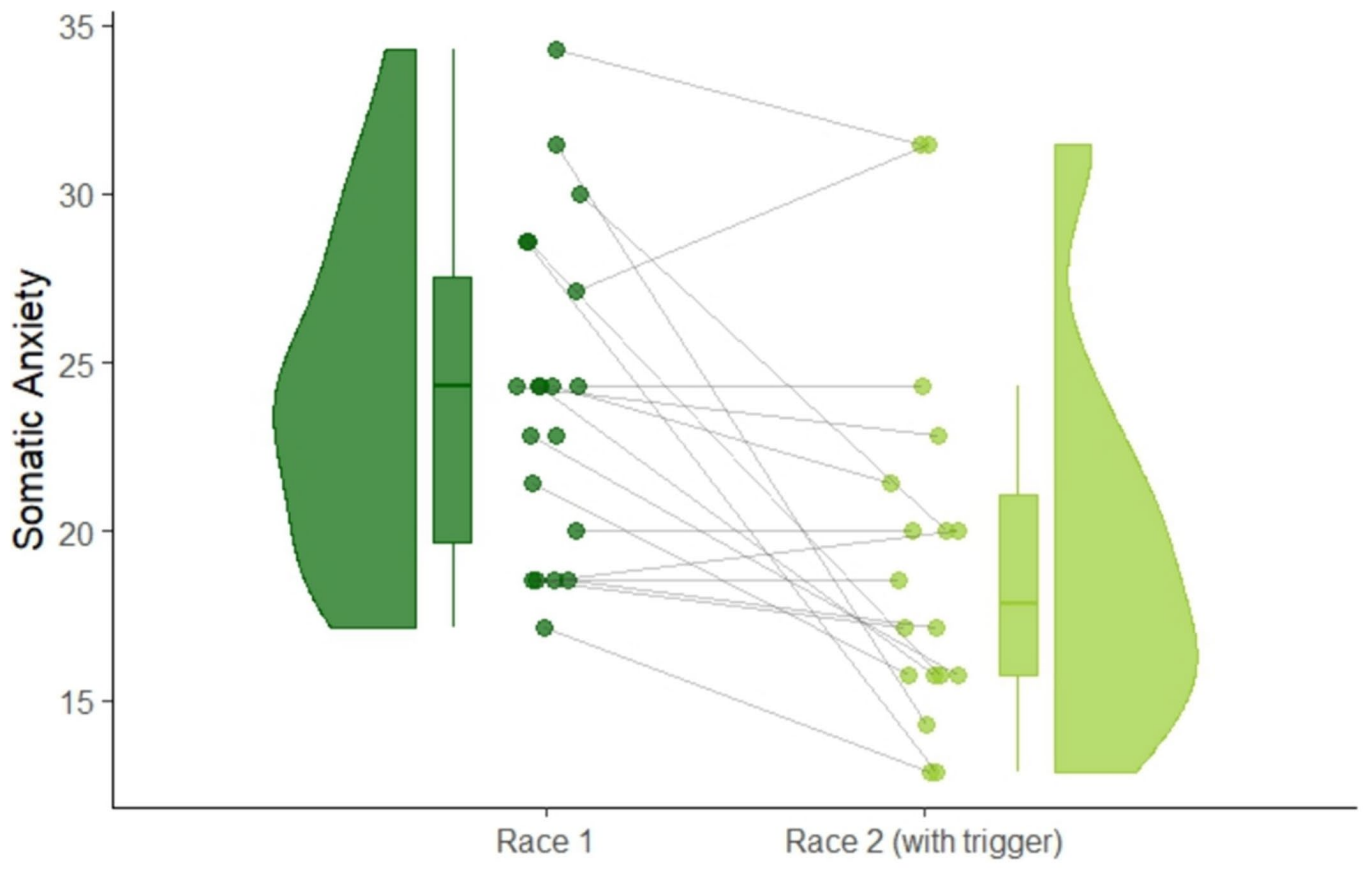
Raincloud plot of within-subject changes of somatic anxiety scores measured via CSAI-2R from race 1 to race 2.

Athletes in the hypnosis group rated their anxiety and self-confidence before each race. Results of the CSAI-2R are shown in Figure 3. Athletes showed significantly less somatic anxiety after listening to the hypnosis audio-intervention, revealed by a reduction of CSAI-2R somatic anxiety scores from 2.4 (SD = 4.81) at the first race to 1.93 (SD = 5.46) at the second race on a 4-point Likert scale (*t*(17) = 3.36, *p* = 0.004, *d_z_* = 0.79). There were no significant changes in cognitive anxiety between races: *t*(17) = 0.88, *p* = 0.43.

**Figure 4:**
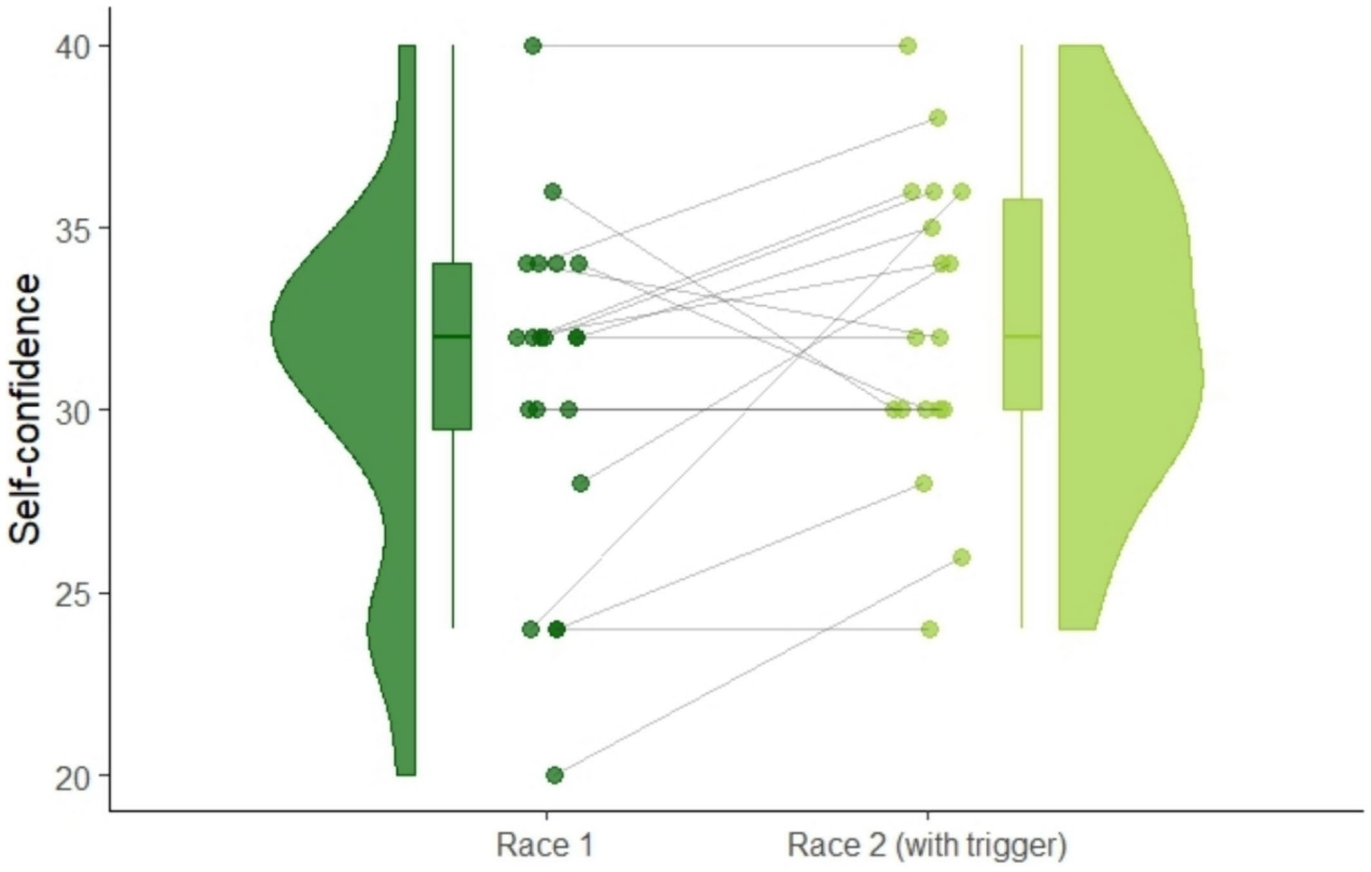
Raincloud plot of within-subject changes of self-confidence scores measured via CSAI-2R from race 1 to race 2.

Self-confidence, shown in Figure 4, increased significantly after listening to the hypnosis audio-intervention, shown by increased CSAI-2R self-confidence scores from 3.07 (SD = 4.74) at the first race to 3.23 (SD = 4.2) at the second race on a 4-point Likert scale: *t*(17) = 1.89, *p* = 0.04 (one-tailed), *d_z_* = 0.45. After finishing the race, athletes filled an additional questionnaire about their subjective feelings of their race performance and feelings immediately before the race start on a 5-point Likert scale ranging from 1 (“I fully agree”) to 5 (“I fully disagree”). There was a significantly higher agreement to the sentence: “I was self-confident before my race start.” changing from 2.23 (SD = 1.0) at the first race to 1.79 (SD = 0.97) at the second race: *t*(17) = 1.93, *p* = 0.04 (one-tailed), *d_z_* = 0.58.

As an exploratory analysis, we investigated gender effects on self-confidence. We found that only female’s CSAI-2R self-confidence scores improved equivalent to 0.58 points on 4-point Likert scale from 2.7 (SD = 0.59) to 3.3 (SD = 0.53) points. The self-confidence scores of the male athletes did not change (first race: *M* = 3.2, SD = 0.36, second race: *M* = 3.2, SD = 0.4).

### Flow state

The scores of the FKS showed no changes between races in flow state (*t*(18) = 0.14, *p* = 0.89) and concerns (*t*(17) = 0.79, *p* = 0.44).

### State resilience and subjective stress

**Figure 5:**
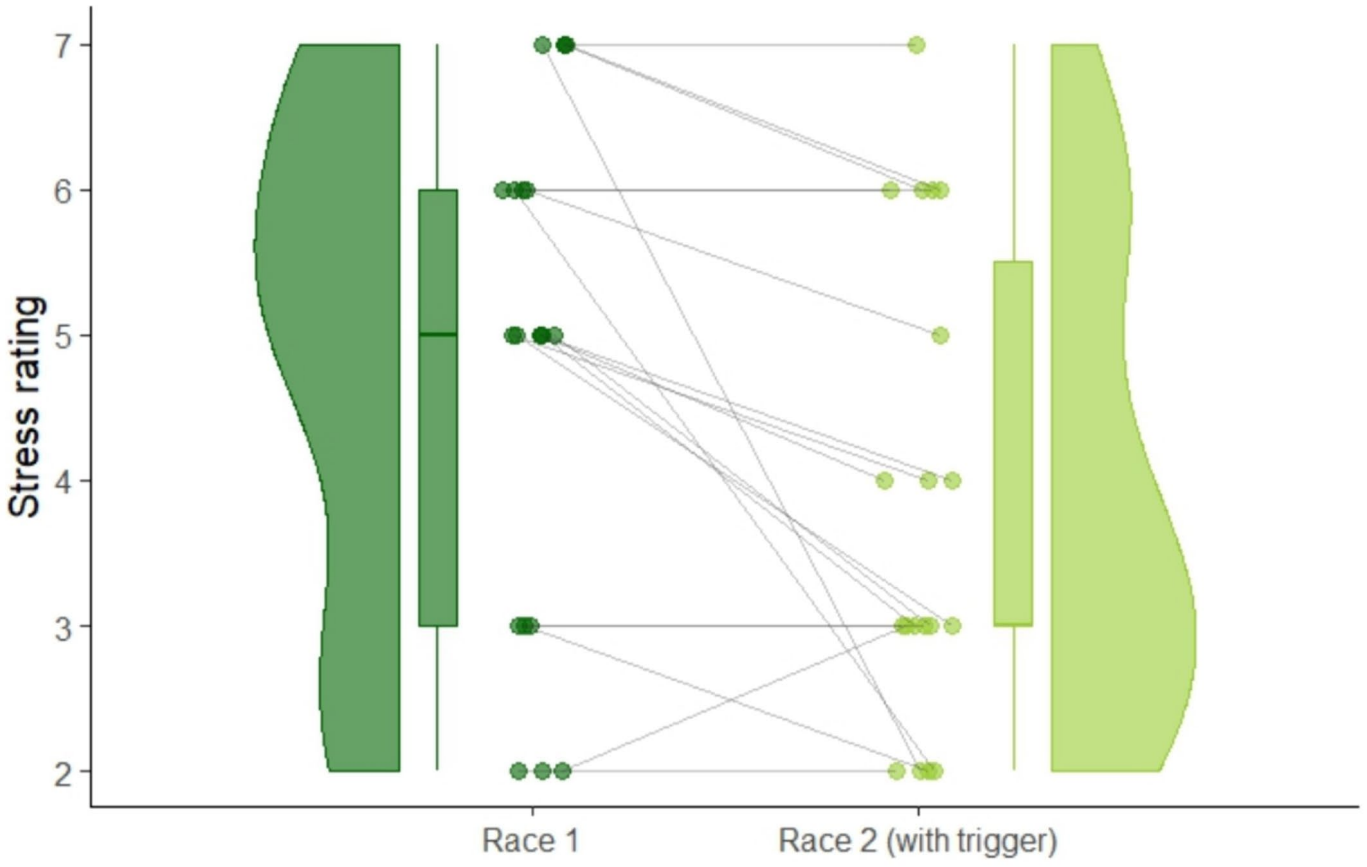
Raincloud plot of within-subject changes of self-reported ratings of stress before the race from race 1 to race 2.

The state resilience questionnaire showed no significant changes from first to second race in state resilience: *t*(18) = 0, *p* = 1. Subjective stress before the race, shown in Figure 5, decreased significantly after participants listened to the hypnosis audio-intervention: *t*(18) = 3.33, *p* = 0.002, showing a large effect size of *d_z_* = 0.76.

### HRV data

We found a significant decrease in heart rate by 3 bpm from first to second race: *t*(18) = 1.75, *p* = 0.049 (one-tailed), *d_z_* = 0.32. There were no significant changes in MEANRR (*t*(18) = 1.99, *p* = 0.06) and RMSSD (*t*(18) = 0.93, *p* = 0.36).

**Figure 6:**
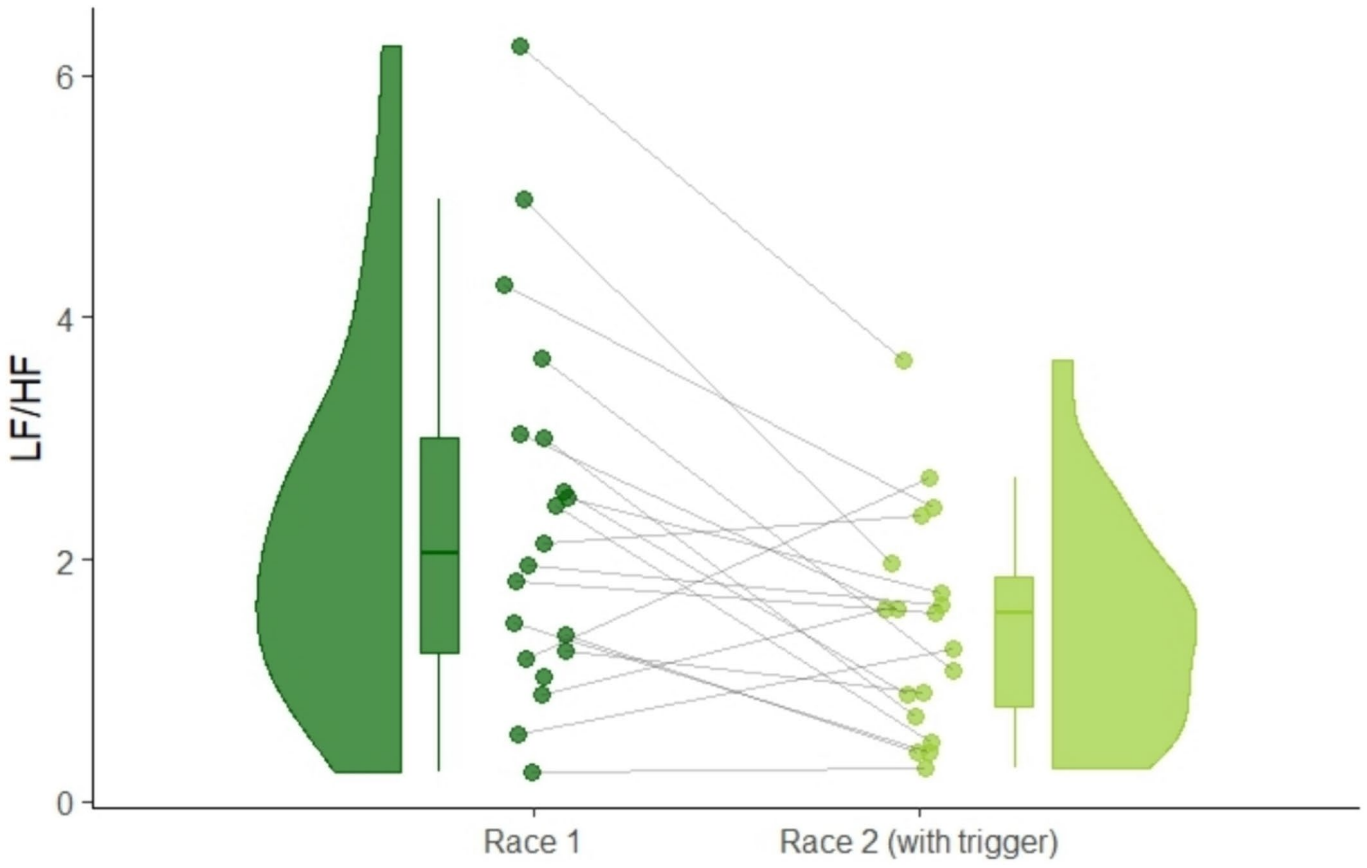
Raincloud plot of within-subject changes of LF/HF ratio from race 1 to race 2 as an indicator of increased parasympathetic activity.

Concerning HRV, we found significant changes in HF_nu_ and LF/HF ratio. There was a significant increase in HF_nu_ from first to second race: *t*(18) = 2.64, *p* = 0.02, *d_z_* = 0.61. In line with that, we also found a significant decrease in LF/HF ratio from first to second race: *t*(18) = 3.26, *p* = 0.004, *d_z_* = 0.75, Figure 6. These results indicate lower sympathetic and higher parasympathetic activity after athletes listened to the hypnosis audio-intervention.

### Evaluation of the audio-hypnosis

Athletes evaluated their perceived feelings of the hypnotic trance and the efficacy of the post-hypnotic trigger after finishing the second race on a 5-point Likert scale ranging from 1 (“I fully agree”) to 5 (“I fully disagree”). All athletes agreed that they were able to engage well with the hypnosis audio-intervention (*M* = 1.42, SD = 0.51). Most of the athletes (*N* = 14) agreed that they were able to use the post-hypnotic trigger well before their race run (*M* = 1.95, SD = 0.78). Almost the same number of athletes (*N* = 12) reported that they felt safer before the race start with using the trigger (*M* = 2.21, SD = 0.71) and again *N* = 12 athletes had the subjective feeling the trigger affected their race performance (*M* = 2.21, SD = 0.85). Athletes reported they felt that pressure has been taken away during the whole race weekend. They reported less self-doubts, were able to smile on race day and had a positive mindset about the upcoming run. One athlete mentioned he had the feeling that some burden has been taken off the shoulders before the start. Another one thanked the research team for providing a method that can be used in any situation where the nervous are getting high. The athletes listened in average 3 times (*M* = 2.94, SD = 1.73) to the audio-hypnosis for 1 week.

## Discussion

In our present study, we present a hypnosis intervention designed to help athletes to start in their mental pole position. We tested the effect of this intervention on athletes’ subjective ratings, physiological measures and performance in two official consecutive downhill races. Athletes of the hypnosis group filled in questionnaires and listened to the hypnosis audio-intervention in between both races, athletes of the control group did not. We expected that our hypnosis intervention reduces competitive anxiety and stress, as well as enhances self-confidence and resilience before the start of the second race. Furthermore, we predicted that athletes in the hypnosis group experience higher levels of flow state during the second race. We also expected changes in HRV parameters that are associated with parasympathetic activity measured 1 h before the race. Finally, we presumed to improve race performance in the hypnosis group.

We found significant reductions of somatic anxiety and stress in the hypnosis group. Athletes also showed significantly enhanced self-confidence in the race following the hypnosis audio-intervention. Athletes’ heart rate variability increased significantly after they listened to the hypnosis audio-intervention, which is a sign of more parasympathetic activity and thus better stress resilience. We did not find higher flow state levels after the hypnosis intervention and race performance did not improve in the hypnosis group compared to the control group. Overall, we conclude that the present study provides a quantitative proof of the positive effects of a hypnosis audio-intervention on elite downhill Mountainbike athletes. Our intervention helps athletes to deal with the high pressure associated with a downhill Mountainbike race. Our findings contribute to the existing literature on the positive effects of hypnosis on sport performance and well-being in sport (Milling and Randazzo, 2016). Combining the setting of two real bike races and the experimental assessment of subjective ratings and physiological data conducted in that setting results in high ecological validity of our findings. Our intervention is thus a promising technique to improve athletes’ mental strength and activating their inner resources of power, pride and flow that gets them in the mental pole position.

### Race performance

We were expecting that our hypnosis audio-intervention increases athletes’ mental strength on the race day and therefore improves race performance in the hypnosis group. The hypnosis group performed better than the control group at both races. This might reflect a selection bias as especially good athletes are highly motivated to improve performance with new techniques (Atkinson and Nevill, 2001). However, we did not find any improvements of race performance in the hypnosis group after they listened to the hypnosis audio-intervention. Results of a downhill Mountainbike race depend on many different variables like physical exertion, track and weather conditions, bike material or competitors (Chidley et al., 2015). Mental strength is one variable to influence race performance. Furthermore, our hypnosis intervention has only been given to the athletes for 1 week. A longer period would give our hypnosis intervention more time to unfold its potential with athletes being able to manifest their mental pole position before races (Schmidt et al., 2024b), integrate it in their training and perhaps improve race performance.

### Competitive anxiety, self-confidence, and flow state

Empirical research showed that competitive anxiety is more detrimental to sports that require coordination and fine motor skills (Mellalieu et al., 2006). downhill Mountainbike adds to those types of sports with requiring high cognitive capacities. Thus, a low level of competitive anxiety and arousal should help athletes reaching their IZOF. With our hypnosis intervention we were able to lower the feelings of somatic anxiety before the race.

The hypnosis audio-intervention did not affect cognitive anxiety levels. Hypnosis elicits changes in individuals on an unconscious or subconscious level, which manifests in emotional and somatic changes (Schmidt et al., 2021). Cognitive changes in turn are conscious, what could explain why cognitive anxiety has not been affected. One of the athletes reported to not remember the personal trigger but suddenly had the desire to listen to one specific song before the race. This exemplary shows that hypnosis works in an un- or subconscious way.

We were able to raise self-confidence levels from first to second race. Our audio-hypnosis included the activation of feelings of pride and happiness before the race, which perhaps leaded to the increase in self-confidence. As self-confidence being one of the most important variables for descending time in downhill Mountainbike an increase of self-confidence is a strong achievement (Chidley et al., 2015). Additionally, an increase in self-confidence and the recall of an optimal performance may serve as a buffer against competitive anxiety and help athletes to deal better with the pressure of a competition (Milling and Randazzo, 2016).

We did not find any changes in flow state. To enter a flow state anxiety and stress levels must be low and on an individual optimal level (Nakamura and Csikszentmihalyi, 2009). We were able to reduce both, stress and anxiety, but the post measurement took place only 1 week after the intervention. This may have been too early to elicit changes in flow state.

### HRV data

Anxiety and competitive stress situations lead to a decrease of vagal activity which can be measured using HRV parameters (Morales et al., 2013). With our hypnosis intervention we tried to reduce anxiety and stress levels and enhance parasympathetic nervous system activity 1 h before the race. By the time we conducted the resting HRV measurement athletes had not started their warm-up yet, so a calm and relaxed mindset is desirable. We showed an increase in HF_nu_ and a decrease of LF/HF ratio before the second race. Hence, athletes felt more relaxed and less stressed leading to higher resilience 1 h before the second race. This is a desirable outcome as elevated stress levels harm mental health and can lead to an increased risk of injury (Ivarsson et al., 2017). Especially downhill Mountainbike athletes are delicate for injuries with downhill Mountainbike being high-risk sport and injury prevention has highest priority (Guszkowska and Boldak, 2010).

### Hypnosis audio-intervention

All athletes listened to the audio-hypnosis themselves at home in a quiet room between the two competitions. After the second race, they rated their subjective experience with the hypnosis session on a 5-point Likert scale in four self-developed sentences, such as “I was able to dive well into the hypnotic trance,” “I had the feeling the hypnotic intervention helped me in my next competition,” “I was able to use the post-hypnotic trigger before my next race” and “I felt safer before the race with the use of my post-hypnotic trigger.” The hypnosis intervention has been accepted well by all athletes. Every athlete was able to engage well with the hypnosis intervention and most of them (*N* = 12) rated the individual trigger as an effective tool before the race start to provide safety and enhance performance. Qualitative feedback given to the experimenter by each athlete after the second race was primarily positive. All but three athletes were sure that the hypnosis intervention was helpful and effective. This subjective feedback shows that this audio-intervention might be an appropriate tool for a majority of downhill Mountainbike athletes.

We did not test hypnotic susceptibility in the athletes. All athletes participated voluntarily in the study. There has been high curiosity, a positive attitude, and high expectations of hypnosis, which has been shown to be important predictors of hypnotic responding (Kirsch et al., 1995). The motivation of all athletes to engage with the hypnosis intervention has been high due to a real race setting. Furthermore, high rapport has been created by recording the audio-hypnosis by Nina Hoffmann. Hence, we expected athletes to be highly susceptible for hypnosis.

Two hypnosis interventions can be classified as efficacious for enhancing sports performance (Milling and Randazzo, 2016). In our study, athletes listened on average three times to the audio-hypnosis (SD = 1.73, range = 1–7). As none of the athletes had experience with hypnosis, many reported easier engagement with the hypnosis intervention when listening the second time. This indicates that a longer period with more hypnosis session may have larger effects. However, we found no significant correlations between the times athletes listened to the hypnosis intervention and somatic anxiety, self-confidence, subjective stress or HRV data.

### Future directions

Our study represents an innovative approach and has high ecological validity. The inclusive nature of our sample population strengthens the external validity of our findings and underscores the relevance of hypnosis as a performance-enhancing tool for a broad spectrum of athletes. The intervention focused on competitive performance in an unconventional sport setting and all effects have been detected in a real downhill Mountainbike race setting. Thus, our intervention provides a robust and effective method for downhill Mountainbike athletes to improve variables that can foster athletic performance. As we conducted a field study, there are a few limitations. First, there has been no control group for all self-recorded data and HRV measurement. Since we acquired data in a real competition setting, we did not want to interfere in athletes race routines of the control group with providing questionnaires and a placebo audio tape. Therefore, we can only compare hypnosis and control group based on their official performance tables. We did the best to rule out possible alternative explanations and used a within-subjects design to measure intraindividual changes. Second, we did not collect individual baseline HRV data outside a competition or in the morning of the competition because of lacking capacity. Third, our post measurement took place only 1 week after the pre measurement. A longer time period between both races where athletes use the hypnotic intervention could have led to measurable performance improvements. Finally, the hypnosis intervention has been provided as an audio recording. An individual hypnosis intervention with each athlete could elicit larger effects and perhaps improve race performance (Robazza & Bortoli, 1995; Pates et al., 2001a; Pates, 2013). Future research is needed to investigate the positive long-term effects of hypnosis interventions on downhill Mountainbike athletes, their mental health and race performance.

## Conclusion and practical implications

The results indicate that our hypnosis audio-intervention is an economic and effective method to induce an optimal performance state in elite downhill mountain bike athletes. We enabled the athletes to harness the positive feelings, enthusiasm and energy they get from mountain biking, which helped them coping with the pressure on race day. The reduced anxiety and increased self-confidence as well as lower subjective and objective stress levels helped the athletes to achieve optimal performance in the competition.

## Data availability statement

The datasets presented in this study can be found in online repositories. The names of the repository/repositories and accession number(s) can be found in the article/supplementary material.

## Ethics statement

The studies involving humans were approved by local ethics committee of the Jena University Hospital, with reference number 2022-2568-BO. The studies were conducted in accordance with the local legislation and institutional requirements. Written informed consent for participation in this study was provided by the participants’ legal guardians/next of kin.

## Author contributions

NH: Writing – review & editing, Writing – original draft, Investigation, Conceptualization. JS: Writing – review & editing, Supervision. BS: Writing – review & editing, Supervision.

## Footnotes

^1^https://www.nina-hoffmann.com/mental
